# PI3K/AKT Signaling Pathway Mediated Autophagy in Oral Carcinoma - A Comprehensive Review

**DOI:** 10.7150/ijms.94566

**Published:** 2024-04-29

**Authors:** Peramaiyan Rajendran, Ramya Sekar, Prabhu Shankar Dhayasankar, Enas M Ali, Salaheldin Abdelraouf Abdelsalam, Sabarinath Balaraman, Biju Vadakkemukadiyil Chellappan, Ashraf M. Metwally, Basem M Abdallah

**Affiliations:** 1 Department of Biological Sciences, College of Science, King Faisal University, Al-Ahsa, 31982, Saudi Arabia.; 2 Centre of Molecular Medicine and Diagnostics (COMManD), Department of Biochemistry, Saveetha Dental College & Hospitals, Saveetha Institute of Medical and Technical Sciences, Saveetha University, Chennai, 600 077, Tamil Nadu, India.; 3 Department of Oral Pathology & Oral Microbiology, Meenakshi Ammal Dental College and Hospital, MAHER, Alapakkam Main Road, Maduravoyal, Chennai-600095, India.; 4 Department of Oral and Maxillofacial Surgery, Meenakshi Ammal Dental College and Hospital, MAHER, Alapakkam Main Road, Maduravoyal, Chennai-600095, India.; 5 Department of Botany and Microbiology, Faculty of Science, Cairo University, Cairo, 12613, Egypt.; 6 Department of Zoology, Faculty of Science, Assiut University, Assiut, 71515, Egypt.; 7 Botany and Microbiology Department, Faculty of Science, Assiut University, Assiut 71516, Egypt.

**Keywords:** oral cancer, PI3K/AKT, autophagy, metastasis

## Abstract

Oral cancer is the most heterogeneous cancer at clinical and histological levels. PI3K/AKT/mTOR pathway was identified as one of the most commonly modulated signals in oral cancer, which regulates major cellular and metabolic activity of the cell. Thus, various proteins of PI3K/AKT/mTOR pathway were used as therapeutic targets for oral cancer, to design more specific drugs with less off-target toxicity. This review sheds light on the regulation of PI3K/AKT/mTOR, and its role in controlling autophagy and associated apoptosis during the progression and metastasis of oral squamous type of malignancy (OSCC). In addition, we reviewed in detail the upstream activators and the downstream effectors of PI3K/AKT/mTOR signaling as potential therapeutic targets for oral cancer treatment.

## 1. Introduction

Head and neck cancer stands as the seventh most common type of cancer world-wide and oral cancer (OC) ranks seventeenth, globally. Every year about 0.66 million new cases and about 0.32 million deaths were recorded 1. In terms of overall population, oral cancer ranks third in India, considering both genders combined. In India, approximately 130,000 new cases of oral cancer are recorded annually, with around 72,000 recorded deaths. India contributes about one-third of the total oral cancer patients, globally 2. Malignancy involving the tongue, floor of the mouth and gingivo-buccal sulcus are the most commonly occurring sub sites of oral cancer. Squamous type of malignancy (OSCC) is the most common type of oral cancer 3. Apart from OSCC the other type of malignancy encountered in oral cavity includes melanoma, lymphoma, sarcoma and salivary gland tumours 4. There is a changing trend in incidence pattern of OSCC. Earlier individuals above 60 years were most commonly affected by the disease, but recently, younger individuals in the middle age group were also affected. Combined genetic and epigenetic factors thought to play a major role in this changing pattern of the incidence 5. Various risk factors including tobacco, alcohol, diet, microbes, inflammation, poor oral hygiene, socioeconomic status and age showed to play an important role as a causative or triggering agent in OSCC. Genetic disorders such as Cowden syndrome and dyskeratosis congenita ought to increase the risk of OSCC 6. Reactive oxygen species (ROS) such as singlet oxygen, hydroxyl radical, superoxide, peroxide ion, nitric oxide and hydrogen peroxide formed as a by-product in various mechanism and promotes carcinogenesis. In addition, ROS induces oxidative damage causing random mutations, that induces various epigenetic modulations 7. Despite tremendous discoveries of various biomarkers for early detection of OSCC, the five-year survivability decreased. The increased death rate relates to diagnosis of the disease at the later stages of the disease 8. PI3K/AKT pathway plays an important role in gene expression, protein synthesis, cell proliferation and survival. Numerous genetic and epigenetic factors were reported to regulate the PI3K/AKT pathway and influence cell proliferation and apoptosis 9. The most common management protocol for OSCC includes surgical resection with or without radiotherapy and chemotherapy. The main chemotherapeutic drugs used in OSCC are cisplatin, 5-fluorouracil, Paclitaxel, Docetaxel, Hydroxyurea. Chemotherapeutic drug resistance occurs commonly due to disordered PI3K/AKT pathway as it mainly controls proliferation and apoptosis 10. This review demonstrated PI3K/AKT as a potential target to induce autophagy in oral squamous cell carcinoma.

## 2. PI3K/AKT pathway

PI3K/AKT acts as a major regulating pathway to maintain the survival of cells in tumour environment with cellular stress 11. This pathway is commonly activated by growth factors such as epidermal growth factor, fibroblastic growth factor and the receptor is of enzymatic type tyrosine kinase (RTK). RTKs are transmembrane proteins, with two different subunits, once the signalling molecules attach to RTK (receptor), it is phosphorylated and support the cross phosphorylation causing dimerization. Dimerization of these receptors activates the PI3Ks. PI3Ks belong to lipid kinase family that has the potency to phosphorylate 3'-OH group in inositol phospholipids 12. The PI3Ks are classified into three different classes based on the protein domains and their regulatory sub-units. Class I group of PI3Ks has catalytic subunits p110 α, β, γ, δ and regulatory subunits p85, p55, p10, p84, p87, p50. Class II group with catalytic subunits PI3KC2 α, β, γ. Class III has Vps34 catalytic subunit and Vps15 regulatory subunit. PI3Ks are very much substrate specific and has distinct mechanism of action and tissue distribution 13, which converts the membrane embedded molecule PIP2 (Phosphatidylinositol-bisphosphate) to PIP3 (Phosphatidylinositol-triphosphate). PTEN (Phosphatase and Tensin homolog) inhibits the process of conversion of PIP2 to PIP3. PIP3 in-turn activates AKT, which helps the cells to proliferate, and divide by modulating GSK3β. Secondly, it also inhibits the apoptotic pathway by modulating MDM2, NF-кB, BAD and FKHR. AKT in turn recruits mTOR that modulates the transcription factors such as S6K1, 4EBP, which further induces protein synthesis, accelerates cell cycle and inhibits the apoptotic initiators 14. AKT accelerates the function of mTOR by inhibiting Tsc1/2 and thereby upregulating Rheb with the help of GTP. mTOR activates the enzyme S6K and downregulates 4EBP1 to promote synthesis of proteins to accelerate DNA repair, upregulate Glut 1, LAT 1, HIF-1α and cyclin D1 and inhibit apoptosis and autophagy 15.

During carcinogenesis, PI3K-AKT pathway activity accelerates to support the nutritional as well as metabolic requirement. It also hyperactivates the cell division, angiogenesis, growth and proliferation and silences the apoptotic and autophagy upregulating proteins. This resulted in consistent tumour growth and decelerated cell death, and thereby, maintaining all the requisites of Hallmarks of cancer 16.

The catalytic subunit of class I PI3K has four isoforms named p110 α, β, δ, ϒ. Although all are functionally distinct and tissue specific, it was observed that mutations of p110 are related to many cancers especially p110α, resulting in abnormal kinase activity. Mutations in PIK3CA, the oncogene coding for p110α seems to be the most commonly mutated gene in various cancer types like liver, colorectal and breast. It is common in prostate cancer, that somatic mutation causing loss of function of the tumour suppressor gene coding PTEN (Phosphatase and tensin homolog) 12,13.

## 3. PI3K/AKT in various cancer

Lung cancer is one of the most common cancers that occurs among men with smoking habits. Mutations of RTKs and EGFR are commonly observed in NSCLC type of lung cancer. In about 67% of the individuals with EGFR mutation, the AKT/mTOR pathway was hyperactivated. Patients with squamous cell carcinoma of the lung were observed with increased mutation in* PIK3CA,* that codes for catalytic subunit, and another component* PIK3R1* which codes for regulatory sub unit of PI3K pathway. Genes including AKT and PTEN were also reported to be mutated in lung cancer patients that modulate the PI3K/mTOR pathway 17.

Breast cancer is the most commonly diagnosed malignancy. Although the diagnosis and treatment protocols show tremendous development towards improving the survival rate, many patients die from drug resistance. The PI3K/AKT/mTOR pathway is strongly associated with drug resistance. Increased mutation of PIK3CA is evident in luminal A subtype, HER2- and ER-positive tumours. Although PTEN mutations were significantly associated with various types of breast cancer, mutations of PIK3CA, AKT and PTEN usually coexist 18. Oesophageal cancers are commonly seen in Asia and rank sixth most common worldwide. Oesophageal squamous cell carcinoma (ESCC) is the most common type of malignancy reported in the oesophagus. PIK3CA seems to be the most common type of mutation in ESCC, with frequencies ranging from 4 to 25%. Single nucleotide polymorphism (SNPs) of AKT1 was reported to play a major role in ESCC incidence, especially among females and non-alcoholics. Other genes which were found to be commonly mutated are PTEN, FRAP1 and mTOR 19. Cervical cancer and human papilloma virus (HPV) are related to tobacco and oral cancer. Oncoproteins E7, E6 of the HPV were reported to modulate PI3K/AKT/mTOR. Patients with PIK3CA mutation of cervical squamous cell carcinoma showed increased survival rate in comparison to those without PIK3CA mutation. Knockdown of DEPTOR and RAGE has been shown to inhibit PI3K/AKT pathway in cervical cancer by decelerating proliferation and inducing apoptosis 20.

Prostate cancer is one of the leading causes of cancer death among men. More than 50% of the castration-resistant prostate tumours had PTEN mutation or deletion. Progression of prostate cancer and multi-drug-resistant tumour was significantly associated with PI3kinase pathway hyperactivation, either due to PTEN modulation or due to hyperactivation of PIK3CA, AKT, mTOR or any other proteins of the pathway 21.

It is evident in gastric cancer that as the severity of the disease increases the frequency of PIK3CA mutation also increases. It was shown by experiment that knockdown of PIK3R3 effectively reduced the progression of tumour. pAKT expression was detected in 78% of gastric cancers. Apart from the above-mentioned proteins and genes, increased activity of GSK3β was significantly correlated to better prognosis 22.

## 4. PI3K/AKT in Head and Neck cancers

Head and Neck cancers (HNC) are one of the most common types of cancers encountered. HNC includes laryngeal cancer, pharyngeal cancer, oesophageal cancer, oral cancer, tongue cancer and other unidentified cancers of head and neck region. Oral squamous cell carcinoma (OSCC) is the most common type of oral cancer. Tobacco consumption habit stands the most common cause of OSCC. Alcohol acts as an adjuvant to tobacco habit. Apart from the tobacco by-products and many synthetic carcinogens such as 4-NQO, various single nucleotide polymorphisms (SNP) were also shown to activate PI3K/AKT pathway 23.

Targeted therapy to inhibit epidermal growth factor (EGFR), one of the ligands of RTKs, was thought to be a promising therapeutic option. However, due to increase in resistance to EGFR therapy, its usage is limited clinically. In OSCC often the PI3K/AKT/mTOR pathway is directly or indirectly activated, targeted therapies to modulate this pathway could cause a ground breaking change in the therapeutic aspect of OC 24. Many proteins are involved in the PI3K/AKT pathway, PTEN plays a very important role in controlling the conversion of PIP2 to PIP3 thereby accelerating growth and division. AKT has four isoforms and each one of them plays a very distinct role in cell survival 25. Roy *et al.*, studied the specific role of isoforms in OC and they concluded that AKT1 and AKT2 are the frequently isoforms hyperexpressed in OC. Silencing of these isoforms resulted in cell cycle arrest at G2-M phase thereby reducing cell survivability by inhibiting survivin, cyclooxygenase-2 (COX-2), cyclin D1 and Bcl-2, the anti-apoptotic protein in OC 26.

Patel *et al.* 2005, profiled the activity of EGFR in OSCC cell line in comparison to normal cell using a modified western blot technique to identify the potential therapeutic targets in PI3K/AKT pathway 27. Matsuo *et al.* 2018, investigated the role of various key proteins of PI3K/AKT pathway such as AKT, mTOR, GSK3β in cervical lymph node metastasis, using immunohistochemistry. Expression pattern of all the three proteins was higher in patients with metastasis in comparison to those individuals without metastasis and normal, and GSK3β was significantly associated as a predictor marker of poor prognosis 28. Georgy *et al.* 2015, studied the potential role of GRHL3 in suppression of HNC. They reported that the GRHL3 silencing triggered the loss of PTEN causing abnormal proliferation resulting in aggressive form of squamous cell carcinoma. Their study concluded that the targeting of GRHL3/GSK3B/c-MYC would be rationally strategic 29. Zhu *et al.* 2014, studied the role of LB1 in enhancing the treatment efficacy of chemotherapeutic drug cisplatin both *in vivo* and *in vitro*. They concluded that the efficacy of chemotherapy with cisplatin and radiotherapy can be effectively increased by LB1 by modulating MDM2, p53 and AKT 30. Hu *et al.* 2013, studied the role of 3p-RNA in inducing apoptosis by activation of RIG-1. In this study, the activation of retinoic acid inducible gene like receptor using viral dsRNA showed to upregulate apoptotic pathway at high doses in comparison to low one 31.

## 5. PI3K/AKT and Autophagy

Autophagy is the process that occurs by the formation of autophagosome. It helps in degradation of proteins and organelles of the cell and the products are recycled to yield raw materials for further metabolism and cell survival 32. Excessive autophagy can result in apoptosis. Autophagy is the process that regulate the turnover of proteins and organelles inside the cell. In case of extreme hypoxia, endoplasmic reticulum stress, oxidative or cellular stress, autophagic process induces necrosis or apoptosis resulting in autophagic death of the cell 33. Autophagy and apoptosis communication aids in identification of dead cell antigen by immune cells as well physiological clearance of dying cells. Autophagy is activated during accumulation of unfavourable or misfolded proteins. Autophagy regulatory genes (Atg), which in-turn is regulated by ULK1/2 complex and class III PI3 kinase complex (Beclin1, vps34 and Ambra1) govern the process of autophagy 34. Autophagy maintains cellular balance by constantly working on selective or bulk degradation of cellular and metabolic waste. Lack of autophagy may cause genotoxicity, while increased autophagy may result in constant remodelling causing increased energy supply and also facilitates escape from immune surveillance and targeted drug therapy 35.

**Figure 1:**
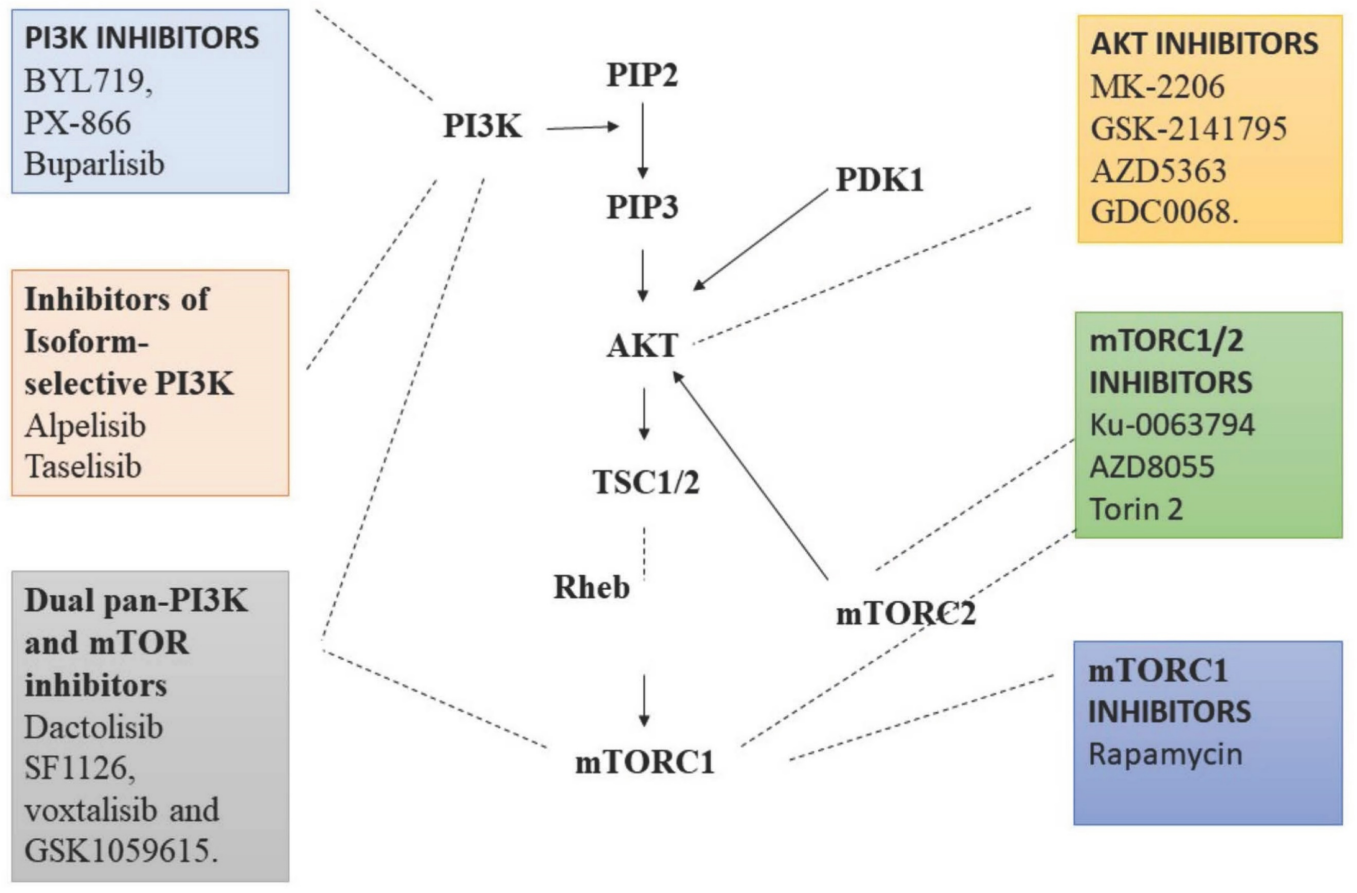
Various Inhibitors that are used as therapeutic agent to target various proteins in PI3/AKT pathway in order to activate autophagy. PI3K: Phosphoinositide-3-kinase; BCR: B-cell antigen receptors; PTEN: Phosphatase and TENsin homolog; PDK1: 3-Phosphoinositide-dependent kinase 1; Akt: Akt serine/threonine kinase family; ULK1: Unc-51 Like Autophagy Activating Kinase 1; ATG: Autophagy Related Gene. (Indicates inhibition…….; Indicates Stimulation →). Created with Biorender in September 2023.

PI3K/AKT/mTOR plays a key role in regulating autophagy. mTOR plays an important inhibitory role in regulating autophagy. Autophagy process is initiated by the formation of phagophore commonly at the juncture of endoplasmic reticulum and mitochondria. Components such as Golgi, other cytoplasmic organelles and plasma membrane also contribute in formation of autophagosomes 36. mTOR phosphorylates autophagy-related protein 13 to prevent the formation of autophagosome. It also promotes the adhesion of ribosome to endoplasmic reticulum which prevents the formation of autophagosome membrane. Thus, upon suppression of PI3K, both AKT and mTOR are blocked and activate autophagy process (Fig. 1) 37.

In the tumorigenic environment the somatic mutations of AKT enhances its activity causing inhibition of proapoptotic signals such as Bad and Bax. Downregulation of Bcl-2-associated death promoter-Bad in turn hyperactivates Bcl-xL-B cell lymphoma extra-large (anti-apoptotic) protein, which further shuts down the apoptosis 38. Similarly, AKT inactivates both Caspases and FOXO-1 (forkhead box protein O1) which regulates the proapoptotic genes like FasL. Along with FOXO, the activity of glycogen synthase kinase 3β will be deregulated and leads to the activation of cyclin D1 as well cyclin-dependent kinase 4 and 6, thereby facilitating the cell entering replication phase. AKT also increases cytoplasmic localization of p27 which plays an important role in metastasis 39. AKT also modulates mTOR along with the above said proteins. AKT inhibits TSC-2 tuberous sclerosis complex-2 by phosphorylation thereby preventing the activation of RHEB (Ras homolog enriched in brain), which eventually leads to activation of mTORC1 mammalian target of rapamycin complex 1 and eukaryotic translation initiation factor 4 complex (eIF4). Activation of mTORC1 and eIF4 accelerates cell cycle, tumour progression, angiogenesis and altered apoptosis 40.

The activation of mTORC1 results in complete activation of AKT by phosphorylation on C-terminal region at serine residues- Ser472, Ser473, Ser 474 of AKT3, AKT1 and AKT2, respectively. mTOR associated proteins including DEP- Dishevelled, Egl-10 and Pleckstrin, mLST- mammalian lethal with SEC13 protein 8 and DEPTOR-domain-containing mTOR-interacting protein signal the mTOR complexes. On the other hand, MAPKAP1-mitogen-activated protein kinase-associated protein 1, RICTOR-Rapamycin-Insensitive Companion on mTOR, PRAS40- proline rich AKT substrate of 40kDa and RAPTOR- regulatory-associated protein of mTOR interact with mTORC1 and mTORC2 to modulate the access to active sites of mTORC1 and mTORC2 41. mTORC1's key role in various cell processes is regulated by the activation of S6, which also modulates translation of 4E-BP1, the binding protein that regulates cell proliferation, protein synthesis, metabolism and apoptosis. The MAPKAP1 along with PI3K protein activates and regulates the function of mTORC2, which further enhances the above-mentioned cell processes 42. Another important function of AKT/mTOR axis is that, it deregulates GSK-3β which helps in glycogen synthesis, modulates fatty acid synthesis by governing ATP citrate lyase and also regulates the proteins involved in glucose transport (PIP5K, AS160) and glycolysis (Hexokinase, 6-phosphofructo-2-kinase/fructose-2, 6-biphosphatase 2 (PFKFB2), Fructose-2) to further enable the suitable environment for tumorigenesis 43.

## 6. Role of ROS in Autophagy

Reactive Oxygen Species (ROS) are a by-product of various cellular metabolic activity. The metabolic activity is higher and continuous in cancerous cells to constantly keep the pace of cellular replication, hence leading to increased production of ROS 44. On the other hand, increased accumulation of ROS in normal or cancerous cell can cause detrimental effects. To combat those effects, the ROS removed from the cell by autophagic machineries. Autophagy in such circumstances not only remove the damaged components of the cell, but also provide the raw materials for mitophagy. Increased nutrients in a cell activates mitochondria resulting in increased ROS production that activates Beclin1 and ATG genes to induce macro-autophagy 45. Beclin1 is bounded to BH3 domain of antiapoptotic molecule Bcl-2 in its inactive state. Increased accumulation of ROS dissociates Beclin1 from Bcl-2 and thus, initiating the process of autophagy. Increased regulation of Beclin1 also leads to apoptosis by binding to the proapoptotic factors in the mitochondrial membrane 46. Apart from the endogenous ROS, those that are derived exogenously such as flavonoids, ionizing radiation can also promote autophagy/apoptosis by activating PTEN and inhibiting PI3K/AKT pathway. ROS also regulate signal that modulate MAPK/JNK, Ras, Raf, ERK, MEK, which ultimately activates autophagy. Therefore, the utility of these interactions in radiotherapy and chemotherapy resistant oral squamous cell carcinoma are reviewed in this article 47.

## 7. PI3K/Akt Autophagy in Oral Squamous Cell Carcinoma

The role of autophagy in tumour progression and metastasis has been frequently investigated in OSCC. Chen *et al.* 2023, studied the efficacy of nanoparticles incorporated hydroxychloroquine by inhibiting autophagy in oral cancer individuals. The study demonstrated that the effect of nanoparticles reducing the scavenging reactive oxygen species helps in success of targeted therapy 48. Vo *et al.* 2021, studied the role of surfactin in inducing cell cycle arrest, apoptosis and autophagy in human OSCC cell line. They reported that surfactin could regulate autophagy and associated with apoptosis and also cause cell cycle arrest 49. Qiu *et al.* 2023, investigated the role of (FBXW7) F-box and WD repeat domain containing 7 in OSCC cell line. They observed, that FBXW7 expression was lessened, which in-turn increased the autophagic activity by accelerating Atg7, Beclin1, BCL2, BAX and BAK. In xenograft tumour model, it was also observed that FBXW7 inhibits cell proliferation and promotes autophagy 50. Borges *et al.* 2023, studied the role of 16 naphthoquinones, which inhibits PKM2 to induce autophagy via PI3K/Akt pathway and its associated apoptosis. It was concluded, that the compound 6a was the most potent drug used in the treatment of cancer and was well tolerated by the animals, extremely selective and had promising pharmacokinetic properties 51. Wen *et al.* 2023, reported that Rho-associated protein kinase (ROCK) inhibitor Y-27632 modulated Akt/mTOR pathway to induce autophagy and further tumour progression. They concluded that Y-27632 could act as a potent therapeutic drug in OSCC 52.

Zhang *et al.* 2023, reported that ITGA6 could act as an excellent prognostic biomarker in OSCC and plays a very important role in tumour progression by modulating mTOR associated autophagy and apoptosis 53. Ma *et al.* 2023, developed ATPscore based on differentially expressed genes in relation to autophagy and identified its regulation pattern. Based on these, the authors identified the suitable genes for autophagy targeted therapy and immunotherapy to increase the survival rate in OSCC individuals 54. Yang *et al.* 2023, investigated the autophagy and apoptotic activity of Antrodia salmonea (AS) in head and neck squamous cell carcinoma cell line. It was observed that AS was a potent inducer of reactive oxygen species mediated autophagy and associated apoptosis 55. Abirami *et al.* 2018, evaluated the expression pattern of p-mTOR in various grades of OSCC in comparison to normal tissue to identify the dyspegulation in PI3K/Akt/mTOR pathway and identified p-mTOR as an effective prognostic marker 56. Kapoor *et al.* 2014, studied the anti-cancer activity of Erufosine by inducing autophagy via modulation of mTOR. Apart from autophagy, the compound showed effective therapeutic effect on the entire PI3K/Akt/mTOR pathway causing cell cycle arrest by downregulation of cyclin D1 and also induced apoptosis 57. Inui *et al.* 2013, studied the expression pattern of p62 and its association with the survival rate by modulating PI3K/Akt pathway 58. Tang *et al.* 2013, studied the endogenous expression of LC3-II in 90 tumour specimens of oral cavity. In this context, high LC3-II expression correlated with poor disease survival 59. Similarly, Sakakura *et al.* 2015, studied the LC3-II expression and correlated the role of immunology in prognosis of OSCC 60.

Wu *et al.* 2014, studied the role of ionizing radiation in inducing autophagy in OSCC cell lines. They also observed that autophagy induced apoptosis via PI3K/Akt was stimulated by ionising radiation 61. Weng *et al.* 2014, also studied the role of Beclin1 in tongue squamous cell carcinoma. They concluded that knockdown of Beclin1 resulted in increased proliferation and invasion of tumour cells due to downregulation of autophagy associated proteins 62. Ahn *et al.* 2011, studied the autophagic activity of Apicidin in OSCC cell lines. Apicidin was found to increase the expression of LC3-II, ATG5 and also to increase the accumulation of acidic vesicular organelles thereby induced autophagy 63 (Table 1).

## 8. Regulation of PI3/Akt through epigenetic modification

The PI3K/AKT signaling pathway not only plays a crucial role in regulating cell survival, proliferation, and apoptosis but also has significant involvement in autophagy, a cellular process that degrades and recycles cellular components. Autophagy is a critical cellular mechanism for maintaining cellular homeostasis, responding to nutrient starvation, and removing damaged organelles or pathogens.

The epigenetic regulation of the PI3K/AKT pathway impacts autophagy, influencing cellular health and disease states by DNA methylation which can regulate the expression of genes involved in the PI3K/AKT pathway and autophagy. The methylation status of the promoter regions of certain autophagy-related genes (ATGs) can influence their expression.

Moreover, the methylation of genes encoding components of the PI3K/AKT pathway can alter its activity, indirectly affecting autophagy. For instance, hypermethylation and subsequent silencing of PTEN (a negative regulator of the PI3K/AKT pathway) can enhance AKT signalling, which typically inhibits autophagy by activating mTOR, a key negative regulator of autophagy. Histone modifications can also influence the expression of genes related to the PI3K/AKT pathway and autophagy. Histone acetylation and methylation can either promote or repress the transcription of specific genes, depending on the specific modification and the site on the histone tails. Acetylation of histones near ATG genes can promote their expression and thus enhance autophagy. Conversely, specific histone deacetylases (HDACs) can suppress autophagy by deacetylating histones near ATG genes, leading to chromatin condensation and gene silencing. Non-coding RNAs, including microRNAs (miRNAs) and long non-coding RNAs (lncRNAs), can regulate the PI3K/AKT pathway and autophagy at the post-transcriptional level. Certain miRNAs can target mRNAs encoding components of the PI3K/AKT pathway or directly target mRNAs of autophagy-related genes, modulating their stability and translation. For instance, miRNAs that inhibit the expression of AKT can indirectly promote autophagy by reducing the activation of mTOR. Similarly, lncRNAs can interact with miRNAs, mRNAs, or proteins to modulate the expression of genes involved in the PI3K/AKT pathway and autophagy, acting as scaffolds, decoys, or guides. The interplay between the PI3K/AKT pathway and autophagy is complex and can be influenced by various epigenetic mechanisms. Dysregulation of these epigenetic processes can lead to altered autophagy, contributing to the pathogenesis of numerous diseases, including cancer, neurodegeneration, and metabolic disorders. Understanding the epigenetic regulation of the PI3K/AKT pathway and autophagy provides insights into potential therapeutic targets for modulating autophagy in disease treatment and prevention.

## 9. PI3K/Akt- a potential target

Oral cancer seems to have the most heterogenous characteristics, both clinically, histologically and have highly variable mutations at genetic level. Despite such heterogenicity, the PI3K/Akt/mTOR pathway was found to be the most commonly modulated 64. This might be due to involvement of number of proteins that regulate major cellular and metabolic activity of the cell via this pathway. Activation of PI3K/Akt pathway seems to activate autophagy due to its effect on mTOR. PI3K/Akt/mTOR pathway has a very complex function and offers several therapeutic targets which mainly include mTOR and PI3K inhibitors. PI3K/Akt can be altered through various factors such as genomic alterations of PIK3CA or ligands such as EGFR, IGR, FGFR, VEGFR, TLR, BCR or PTEN - known as the upstream activators. The downstream effectors such as GSK3, mTOR, FOXO, MDM2, Akt or Beclin1 can alter the pathway. PI3K/Akt also modulates other pathways such as Ras, Raf, NF-кB, thence mutations in PI3K/Akt may further modulate other pathways positively enhancing the tumour environment 43. PI3K/Akt also regulates metabolic process through glycolytic pathway, inflammation, immunity, and angiogenesis. PI3K/Akt (PIP3, β-catenin, E-cadherin) plays an important role in metastasis by regulating the crosstalk between epithelium and mesenchyme, causing Epithelial Mesenchymal Transition 65.

**Figure 2:**
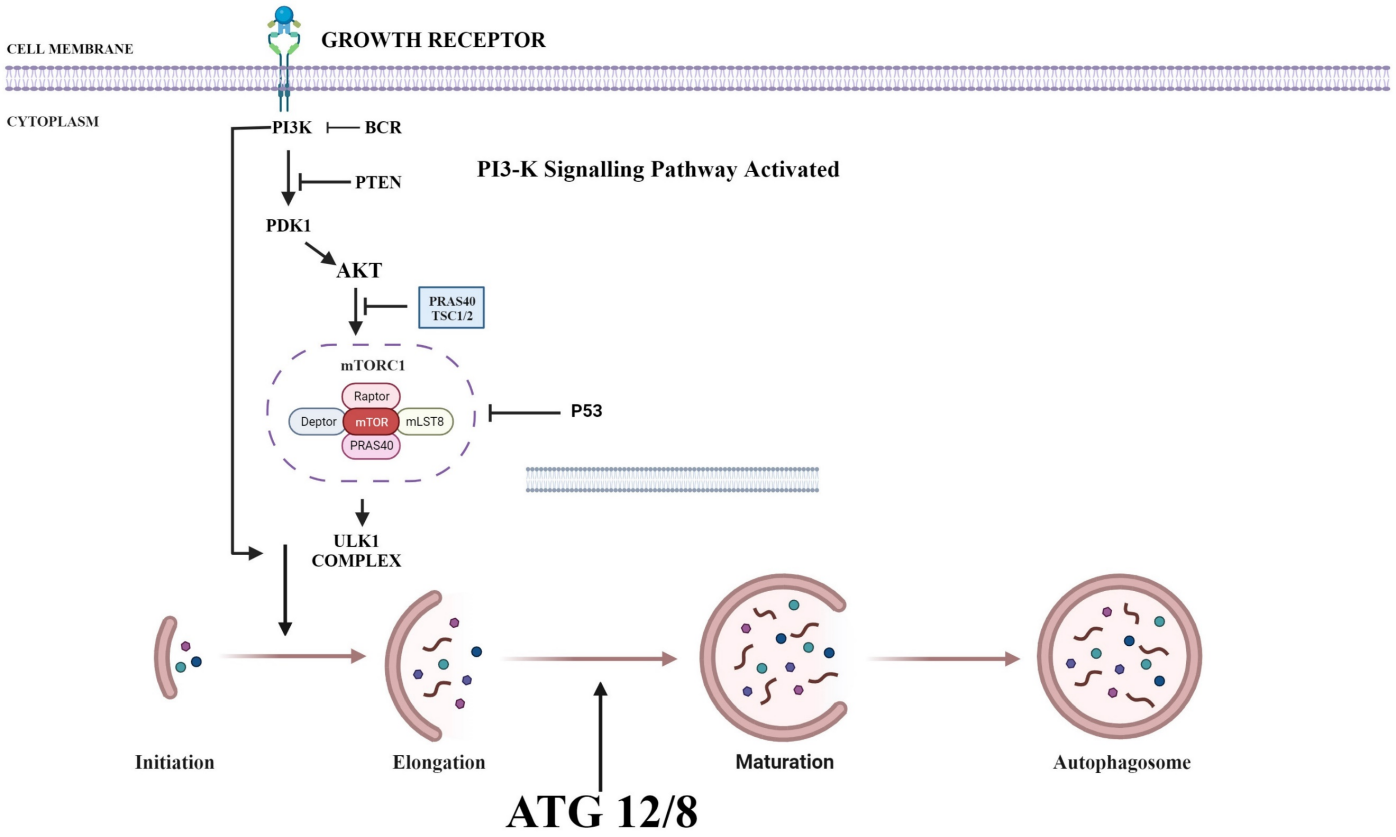
Targets on PI3K/Akt that inhibit the function of various proteins in the pathway to decelerate cell proliferation, angiogenesis, epithelial-mesenchymal transition and promote autophagy-apoptosis axis. Created with Biorender in September 2023.

Various therapies target PI3K/Akt pathway, including the PI3K inhibitors such as Pan-PI3K inhibitors that targets p110 subunits of class IA PI3Ks. For examples, BYL719, PX-866 and buparlisib, which have better anti-tumour activity with less toxicity 66. Inhibitors of isoform-selective PI3K, these therapeutic agents exhibit more specificity to tumour types with less off-target toxicity in comparison to pan-PI3K inhibitors. These includes alpelisib and taselisib 67. Dual pan-PI3K and mTOR inhibitors exert improved effect, as they inhibit both mTOR and PI3K. The combined therapeutic effect helps in the inhibition of entire PI3K/Akt/mTOR pathway. For examples, dactolisib, SF1126, voxtalisib and GSK1059615. Besides their efficacy they are less specific and exert more toxic effects, but they inhibit p110 subunit completely and prevents the feedback mechanism by mTORC1 68. Similarly, Akt inhibitors are currently under trial, including compounds like MK-2206, GSK-2141795, AZD5363 and GDC0068. These act on all three subunits of Akt. mTORC1 and mTORC2 inhibitors are very promising and hotspot therapeutic targets under research 69. Rapamycin are 1^st^ generation mTOR inhibitors which bind to FKBP-12 and mTOR to form a complex, and inhibits the phosphorylation of mTORC1 S6K1 resulting in down streaming of mTORC1 activity. Although these drugs are very promising, varied doses of Rapamycin are required for individuals to visualise the expected outcome 70. ATP-competitive mTOR inhibitors are a group of compounds that act by blocking the active sites of mTOR kinase thereby completely preventing the phosphorylation of Akt. AZD8055 is a recent ATP-competitive mTOR inhibitor, which has both tumour suppressing activity and induces autophagy. It seems to be more potent in rapamycin resistant tumours also 71. In addition, many combination strategies were employed to deliver better outcome. PI3K/Akt/mTOR inhibitors along with GFR inhibitors, MAPK inhibitors and in combination with chemotherapy as well radiotherapy is being tried in drug resistance individuals (Fig. 2) 72.

## 10. Promises and Challenges

Chemotherapy in OSCC causes increased systemic toxicity, which necessitates a more personalised and targeted treatment. Cetuximab, an anti-EGFR targeted drug is the only approved agent for treating squamous cell carcinoma of head and neck region 73. It shows improved survival rate in comparison to standard chemotherapy regimen. Despite its promising results, EGFR resistance seems to be a concerning obstacle. A large number of studies has been conducted to improve the survival rate of OSCC individuals by modulating PI3K/Akt/mTOR associated autophagy 74. Only few drugs such as cisplatin that induce autophagy are licensed for use in oral cancer despite the invention of many compounds due to many contraindications. Many natural compounds, such as areca nut extract 75, curcumin 76, Carfilzomib and ONX 0912 77, G15 78, Tetrandrine 79, Sulfasalazine 80 and other synthetic components 81-84 have been shown to modulate autophagy and associated apoptosis. These effects lead to decreases in tumour growth and increases in survival rate. However, no effective drug has been identified due to the drawback that increased activation of PI3K/Akt causes increased resistance to chemotherapies. On the aspect of immunotherapy, inhibitors of immune checkpoints PD-1, PD-L1 and CTLA-4 are the most successful therapeutic targets. These immune checkpoints are majorly governed by PI3K/Akt. Yet, many cancers such as lung, ovary, breast, prostate have acquired multi-drug resistance (MDR). MDR seems to be the major reason for tumour progression and treatment failure. Hyperactivation of PI3K/Akt could be the major reason for MDR causing poor survival rate. Thereby, targeted therapies must be used with caution without exploitation 85.

## 11. Conclusion

The field of theranostics is a hotspot field of research. Despite the huge effort made in this field, the prognosis of OSCC individuals is very poor and their survival rate seems to be very low in comparison to other malignancies. Tailored treatment modules with less toxicity and better outcomes are need. To meet these requirements, more targeted therapies should be identified with caution, as this could be a double-edged sword. Appropriate targets belonging to the PI3K/Akt/mTOR pathway are highly favourable to achieve better pharmacokinetic results.

## Figures and Tables

**Table 1:** The role of different compounds, genes and proteins in regulating autophagy via PI3K/Akt/mTOR signalling.

S.NO	COMPOUND/ PROTEIN/ GENE STUDIED	METHOD	FUNCTION	REF
**1**	Co-Fc coated nanoparticles	CAL-27 Cell line	Safe and effective delivery system	48
**2**	Surfactin	SCC4 & SCC25 Human OSCC Cell line	Effectively induce apoptosis via autophagy	49
**3**	FBXW7	CAL27 Xenograft tumour model	Promotes autophagy	50
**4**	lawsone, arylaldehydes, and benzylamine	OSCC4, OSCC9, OSCC25 Cell line	Compound 6a was found to be the most potent autophagic agent	51
**5**	Y-27632	Tca8113 and CAL-27 Cell line	Potent ROCK inhibitor	52
**6**	ARG	OSCC tissue	CCL2, CDKN2A, CTSB, CTSD, CXCR4, ITGA6, MAP1LC3A, MAPK3, PARP1, and RAB11A. ITGA6 identified as efficient biomarker	53
**7**	SRPX	FaDu & CAL-27 Cell line	Application of autophagy targeted therapy with immunotherapy in OSCC could be of more therapeutic value	54
**8**	Antrodia salmonea	FaDu Cell line	Antrodia salmonea is a potent anti-tumour agent.	55
**9**	p-mTOR	OSCC tissue	Potent prognostic marker	56
**10**	Erufosine	CAL-27, FaDu, SCC 9, SCC 25 Cell line	Potent inducer of autophagy and apoptosis.	57
**11**	p62/SQSTM1	OSCC Tissue	Act as an early indicator of carcinogenesis and multi-drug resistance.	58
**12**	LC3	OSCC Tissue	LC3 expression indicates poor survival rate.	59
**13**	LC3, p62/SQSTM1, Beclin-1	OSCC Tissue	Expression of proteins LC3, p62/SQSTM1,Beclin-1 indicates poor prognosis.	60
**14**	LC3-II	OSCC OC3, SAS Cell line	Irradiation could increase the autophagic effect in tumour cells.	61
**15**	Beclin 1	TSCC, SCC9, SCC15 Cell line	Potential target for tongue squamous cell carcinoma	62
**16**	Apicidin	YD-8, YD-10B Cell line	Potent inducer of autophagy and apoptosis	63
